# Base-Resolution Sequencing Methods for Whole-Transcriptome Quantification of mRNA Modifications

**DOI:** 10.1021/acs.accounts.3c00532

**Published:** 2023-12-11

**Authors:** Li-Sheng Zhang, Qing Dai, Chuan He

**Affiliations:** † Department of Chemistry, The University of Chicago, Chicago, Illinois 60637, United States; ‡ Howard Hughes Medical Institute, The University of Chicago, Chicago, Illinois 60637, United States; § Department of Chemistry, The Hong Kong University of Science and Technology (HKUST), Kowloon 999077, Hong Kong SAR, China; ∥ Division of Life Science, The Hong Kong University of Science and Technology (HKUST), Kowloon 999077, Hong Kong SAR, China

## Abstract

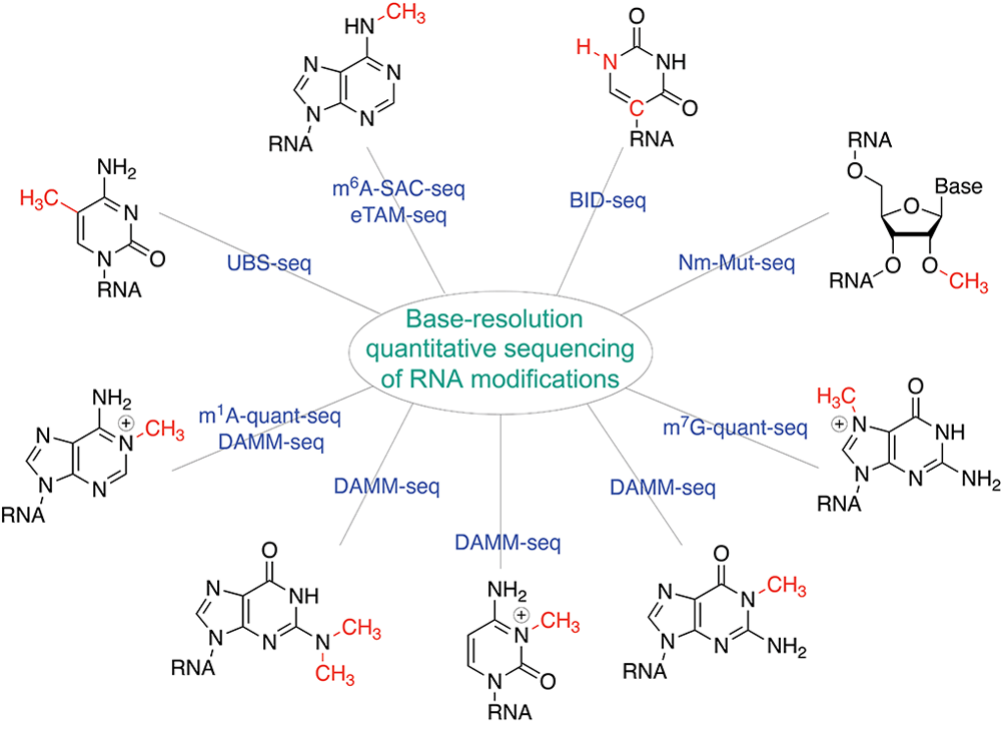

RNA molecules are not merely a combination of four bases of A, C, G, and U. Chemical modifications occur in almost all RNA species and play diverse roles in gene expression regulation. The abundant cellular RNAs, such as ribosomal RNA (rRNA) and transfer RNA (tRNA), are known to have the highest density of RNA modifications, which exert critical functions in rRNA and tRNA biogenesis, stability, and subsequent translation. In recent years, modifications on low-abundance RNA species in mammalian cells, such as messenger RNA (mRNA), regulatory noncoding RNA (ncRNA), and chromatin-associated RNA (caRNA), have been shown to contain multiple different chemical modifications with functional significance.

As the most abundant mRNA modification in mammals, *N*^6^-methyladenosine (m^6^A) affects nearly every stage of mRNA processing and metabolism, with the antibody-based m^6^A-MeRIP-seq (methylated RNA immunoprecipitation sequencing) followed by high-throughput sequencing widely employed in mapping m^6^A distribution transcriptome-wide in diverse biological systems. In addition to m^6^A, other chemical modifications such as pseudouridine (Ψ), 2′-*O*-methylation (N_m_), 5-methylcytidine (m^5^C), internal *N*^7^-methylguanosine (m^7^G), *N*^1^-methyladenosine (m^1^A), *N*^4^-acetylcytidine (ac^4^C), etc. also exist in polyA-tailed RNA in mammalian cells, requiring effective mapping approaches for whole-transcriptome profiling of these non-m^6^A mRNA modifications. Like m^6^A, the antibody-based enrichment followed by sequencing has been the primary method to study distributions of these modifications. Methods to more quantitatively map these modifications would dramatically improve our understanding of distributions and modification density of these chemical marks on RNA, thereby bettering informing functional implications. In this Account, aimed at both single-base resolution and modification fraction quantification, we summarize our recent advances in developing a series of chemistry- or biochemistry-based methods to quantitatively map RNA modifications, including m^6^A, Ψ, m^5^C, m^1^A, 2′-*O*-methylation (N_m_), and internal m^7^G, in mammalian mRNA at base resolution. These new methods, including m^6^A-SAC-seq, eTAM-seq, BID-seq, UBS-seq, DAMM-seq, m^1^A-quant-seq, Nm-Mut-seq, and m^7^G-quant-seq, promise to conduct base-resolution mapping of most major mRNA modifications with low RNA input and uncover dynamic changes in modification stoichiometry during biological and physiological processes, facilitating future investigations on these RNA modifications in regulating cellular gene expression and as potential biomarkers for clinical diagnosis and prognosis. These quantitative sequencing methods allow the mapping of most mRNA modifications with limited input sample requirements. The same modifications on diverse RNA species, such as caRNA, ncRNA, nuclear nascent RNA, mitochondrial RNA, cell-free RNA (cfRNA), etc., could be sequenced using the same methods.

## Key References

- Hu, L.; Liu, S.; Peng, Y.; Ge, R.; Su, R.; Senevirathne, C.; Harada, B. T.; Dai, Q.; Wei, J.; Zhang, L.-S.; Hao, Z.; Luo, L.; Wang, H.; Wang, Y.; Luo, M.; Chen, M.; Chen, J.; He, C. m^6^A RNA modifications are measured at single-base resolution across the mammalian transcriptome. *Nat. Biotechnol*. **2022**, *40*, 1210–1219.^1^ m^6^A-selective allyl chemical labeling and sequencing (m^6^A-SAC-seq) directly reads out m^6^A sites as mutation signatures and, for the first time, achieves the whole-transcriptome base-resolution quantification of m^6^A methylations at diverse motif contexts unbiasedly.
- Dai, Q.; Zhang, L.-S.; Sun, H.-L.; Pajdzik, K.; Yang, L.; Ye, C.; Ju, C.-W.; Liu, S.; Wang, Y.; Zheng, Z.; Zhang, L.; Harada, B. T.; Dou, X.; Irkliyenko, I.; Feng, X.; Zhang, W.; Pan, T.; He, C. Quantitative sequencing using BID-seq uncovers abundant pseudouridines in mammalian mRNA at base resolution. *Nat. Biotechnol*. **2023**, *41*, 344–354.^2^ Bisulfite-induced deletion sequencing (BID-seq) directly maps pseudouridines (Ψ) as deletion signatures without motif bias and, for the first time, achieves the whole-transcriptome quantification of Ψ modifications at single-base resolution.
- Dai, Q.; Ye, C.; Irkliyenko, I.; Wang, Y.; Sun, H.-L.; Gao, Y.; Liu, Y.; Beadell, A.; García, J. P.; Goel, A.; He, C. Ultrafast bisulfite sequencing for efficient and accurate 5-methylcytosine detection in DNA and RNA. *Nat. Biotechnol*. In Press.^3^ Ultrafast BS sequencing (UBS-seq) employs the new chemistry with a high bisulfite concentration and a high reaction temperature, to improve C-to-U conversion efficiency and reduce RNA damage, outperforming all of the reported BS conditions in terms of lower background and higher sensitivity in quantitatively detecting RNA m^5^C sites transcriptome-wide.
- Zhang, L.-S.; Xiong, Q.-P.; Peña Perez, S.; Liu, C.; Wei, J.; Le, C.; Zhang, L.; Harada, B. T.; Dai, Q.; Feng, X.; Hao, Z.; Wang, Y.; Dong, X.; Hu, L.; Wang, E.-D.; Pan, T.; Klungland, A.; Liu, R.-J.; He, C. ALKBH7-mediated demethylation regulates mitochondrial polycistronic RNA processing. *Nat. Cell Biol*. **2021**, *23*, 684–691.^4^ Demethylation-assisted multiple methylation sequencing (DAMM-seq) simultaneously detects m^1^A, m^1^G, m^3^C, and m^2^_2_G methylations as mutation signatures in one sequencing run, enabling the base-resolution quantitative mapping of these four methylations with low RNA input.

## Introduction

**Figure 1:**
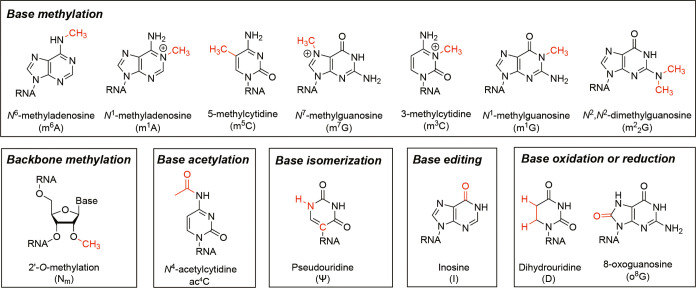
Major RNA modifications in mammalian cells.

Chemical modifications exist in almost all RNA species of mammalian cells, playing critical roles in regulating gene expression. These RNA modifications mainly include base methylation (*e.g*., *N*^6^-methyladenosine (m^6^A), *N*^1^-methyladenosine (m^1^A), *N*^7^-methylguanosine (m^7^G), 5-methylcytidine (m^5^C), *N*^3^-methylcytidine (m^3^C), *N*^1^-methylguanosine (m^1^G), *N*^2^,*N*^2^-dimethylguanosine (m^2^_2_G)), backbone methylation (2′-*O*-methylation (N_m_)), base acetylation (*e.g*., *N*^4^-acetylcytidine (ac^4^C)), base isomerization (*e.g*., pseudouridine (Ψ)), base editing (*e.g*., inosine (I)), and base oxidation/reduction (*e.g*., dihydrouridine (D), 8-oxoguanosine (o^8^G); Figure 1). Abundant noncoding RNA species such as rRNA and tRNA are known to possess dense chemical modifications^5^ that impact their biogenesis, local structure, and stability and modulate the subsequent translation. High modification stoichiometry (modification fraction) is observed at most modified sites in mammalian rRNA and tRNA. The high abundances of rRNA and tRNA allow functional study of their RNA modifications using diverse approaches including quantification of the modification site and stoichiometry with mass spectrometry.^6^

Besides rRNA and tRNA, RNA modifications are also present in low-abundance RNA species, such as mRNA, regulatory noncoding RNA, and chromatin-associated RNA (caRNA), which could not be investigated at each modified site through conventional biochemical approaches. The next-generation sequencing technology offers an opportunity to map the global distributions of these mRNA modifications.^7,8^ Using mRNA purified from human cells as an example, mass spectrometry has revealed the presence of different mRNA modifications, including m^6^A, Ψ, 2′-*O*-methylation, m^5^C, m^1^A, internal m^7^G, ac^4^C, etc. To unveil the biological functions of these mRNA modifications, an ideal sequencing method would not only uncover the location of individual RNA modification transcriptome-wide but also the stoichiometric information at each modified site. In the past several years, our laboratory has been working on developing such quantitative base-resolution methods that allow the evaluation of modification stoichiometry at individual modified sites for almost all major mRNA modifications. Here, we summarize m^6^A-SAC-seq^1^ and eTAM-seq^9^ for m^6^A; BID-seq for Ψ;^2^ UBS-seq for m^5^C;^3^ DAMM-seq^4^ for one-pot sequencing of m^1^A, m^3^C, m^1^G, and m^2^_2_G; m^1^A-quant-seq^10^ for m^1^A; N_m_-Mut-seq^11^ for N_m_; and m^7^G-seq and m^7^G-quant-seq for internal m^7^G^12,13^ in this Account.

### *N*^6^-Methyladenosine (m^6^A)

Among all internal mRNA modifications in mammals, m^6^A is the most abundant, exhibiting ∼0.4–0.6% m^6^A/A abundance in mRNA.^14,15^ m^6^A affects almost every aspect of mRNA processing and metabolism, impacting a variety of biological processes in a living cell.^5,7^ m^6^A methylation on mammalian mRNA is mediated by the METTL3/METTL14 methyltransferase complex as the main m^6^A “writer” protein.^16^ FTO and ALKBH5 were identified by our laboratory as the m^6^A “eraser” proteins in 2010 and 2013,^17,18^ respectively, which reverse m^6^A methylation. Proteins that preferentially bind m^6^A, or “reader” proteins, were also discovered, with YTHDF1–3^19−21^ and YTHDC1–2^22−24^ possessing an evolutionally conserved YTH domain that binds selectively to m^6^A. These proteins bind m^6^A-methylated RNA and regulate mRNA stability, translation, pre-mRNA splicing, nuclear export, and other biological processes. The discovery of m^6^A “writer,” “eraser,” and “reader” proteins largely promote the recent explosion of m^6^A epitranscriptome research; the application of next-generation sequencing technology to map mRNA m^6^A distribution has been an indispensable part in nearly every study related to m^6^A biology.

For over a decade, the antibody-based m^6^A-seq or m^6^A-MeRIP-seq strategy^14,15^ has been a prevalent method to study the distribution of m^6^A methylations at 100–200 nucleotide (nt) resolution. Relying on the anti-m^6^A antibody, miCLIP^25^ and m^6^A-LAIC-seq^26^ provided updated versions. However, these antibody-based methods were unable to provide single-base resolution, m^6^A stoichiometry, nor the sensitivity to compare m^6^A methylation dynamics. MAZTER-seq^27^ and m^6^A-REF-seq,^28^ exploring MazF RNase, selectively cleave RNA at ACA motif without m^6^A methylation. Although this approach could detect m^6^A methylation within the ACA motif, it only accounts for around 15% of the overall m^6^A sites in mRNA and lacks sensitivity. DART-seq,^29^ m^6^A-SEAL,^30^ and m^6^A-label-seq^31^ have also been reported, but they either lack stoichiometric information at m^6^A-modified sites or cannot be applied transcriptome-wide.

**Figure 2:**
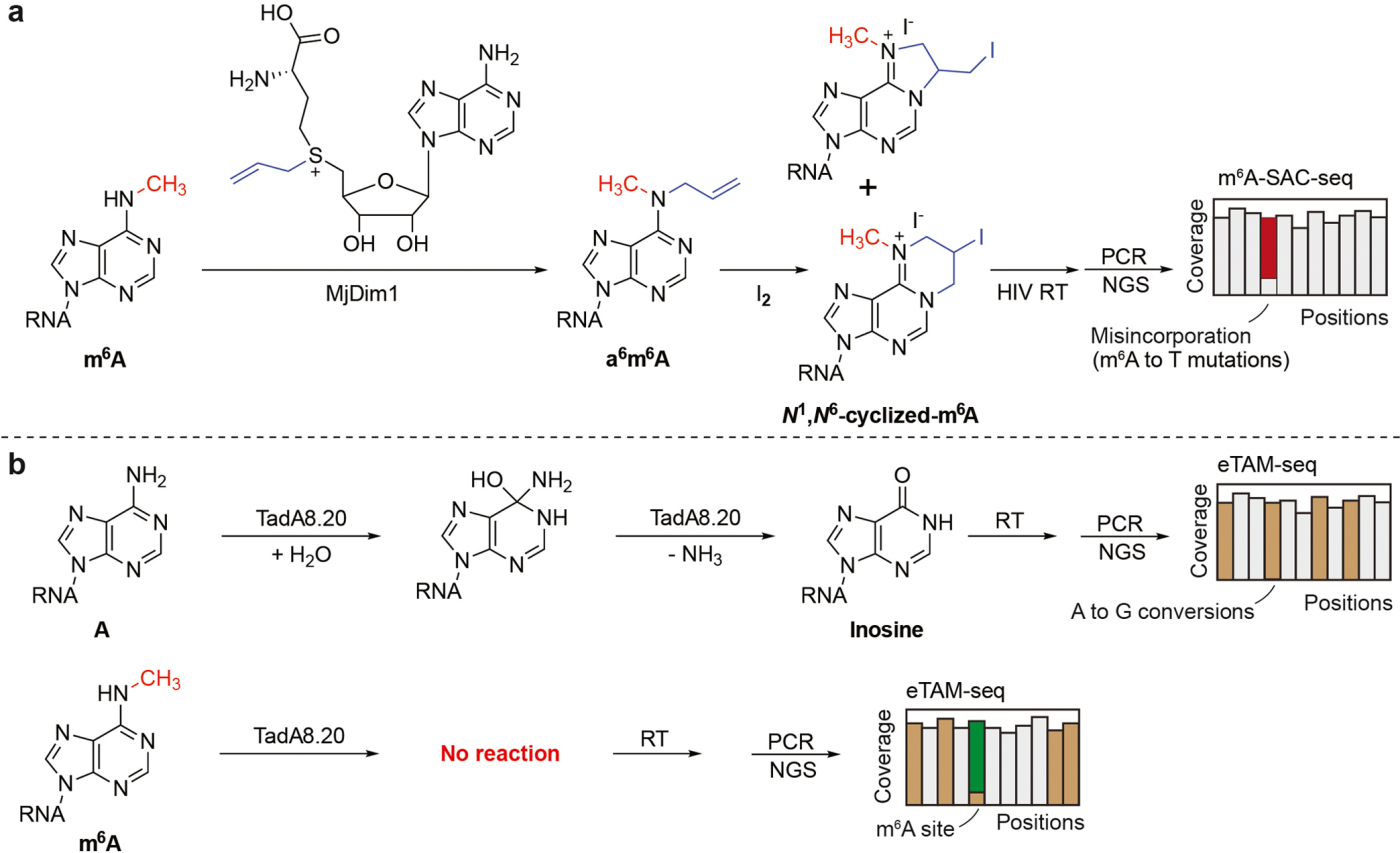
Base-resolution quantitative m^6^A-SAC-seq and eTAM-seq for mapping m^6^A modification in mammalian mRNA. (a) Quantitative m^6^A-SAC-seq maps internal m^6^A sites as misincorporation signatures. (b) Quantitative eTAM-seq induces A to G conversion at unmethylated A sites instead of m^6^A sites.

We have recently reported m^6^A-selective allyl chemical labeling and sequencing (m^6^A-SAC-seq) that reads out m^6^A as mutated signals.^1^ Our approach is based on the unique activity of the *Methanocaldococcus jannaschii* homologue MjDim1, which is known to convert A to m^6^A and then m^6^A to m^6^_2_A.^32^ We found that this enzyme can employ a chemically modified allylic-SAM to install an allyl group at the N6 position of m^6^A. With the allylic-SAM as the cofactor, MjDim1 exhibited a ∼10-fold preference for m^6^A over A in the allyl group transfer reaction, converting m^6^A into allyl-modified m^6^A (*N*^6^-allyl,*N*^6^-methyladenosine, a^6^m^6^A; Figure 2a).^1^ To further induce misincorporation signatures at the generated a^6^m^6^A sites, the subsequent I_2_ treatment converts a^6^m^6^A and a^6^A into the corresponding *N*^1^,*N*^6^-ethanoadenine and *N*^1^,*N*^6^-propanoadenine derivatives, respectively, which can be read out as misincorporation signatures by human immunodeficiency virus 1 (HIV-1) reverse transcriptase (HIV RT; Figure 2a). HIV RT generated ∼10-fold higher mutation rates at the cyclized a^6^m^6^A sites (m^6^A sites) than the cyclized a^6^A sites (unmodified A sites) in almost all sequence contexts in NNm^6^ ANN versus NNANN. Meanwhile, HIV RT could also read through cyclized NNa^6^m^6^ ANN without noticeable RT stops. Therefore, the MjDim1-catalyzed allyl transfer using allylic-SAM shows a ∼10-fold preference for m^6^A over A, and the cyclized a^6^m^6^A adduct (generated from m^6^A) induces another ∼10-fold higher misincorporation rate than the cyclized a^6^A adduct formed from unmodified A. Overall, m^6^A-SAC-seq offers a ∼100-fold higher selectivity at m^6^A over unmodified A sites^1^. Before MjDim1 labeling, RNA fragments could also be split into two sections, “untreated” and “FTO treated”, in which the FTO treatment erases a large portion of mRNA m^6^A as a background control to further eliminate false positives.

m^6^A-SAC-seq maps internal m^6^A methylomes at base resolution, and the misincorporation rates readout at each modified site could be used for assessing the m^6^A modification stoichiometry. By applying m^6^A-SAC-seq to the mixtures of synthetic NNm^6^ANN and NNANN oligo probes in different ratios, we established a spike-in calibration system for each sequencing library and monitored m^6^A modification fractions transcriptome-wide through computation. The initial m^6^A-SAC-seq protocol starts with ∼30 ng of poly(A) RNA or rRNA-depleted RNA. After further optimization of library preparation and bioinformatic pipelines, the latest m^6^A-SAC-seq starts with ∼2 ng of poly(A) RNA with high reproducibility,^33^ revealing ∼30 000–130 000 m^6^A sites that overlap well with m^6^A profiles obtained by antibody-based approaches. For the first time, m^6^A-SAC-seq demonstrated the quantitative base-resolution maps of internal m^6^A modifications at diverse motif contexts unbiasedly, and set the technological basis for sensitively monitoring m^6^A dynamics in diverse biological processes.

As a complement sequencing tool to m^6^A-SAC-seq, which involves enzymic reactions targeting m^6^A bases over unmodified adenosines, through collaboration we have also helped develop evolved TadA-assisted *N*^6^-methyladenosine sequencing (eTAM-seq).^9^ eTAM-seq employs highly efficient global adenosine deamination of unmethylated A sites mediated by TadA8.20 and reads out these unmodified sites as guanosines (G) in next-generation sequencing (NGS) data, while m^6^A is resistant to this enzymic deamination and thus is still read as A (Figure 2b). In this way, eTAM-seq achieves transcriptome-wide, base-resolution detection and quantification of m^6^A. It uncovered ∼35 000 m^6^A-modified sites in HeLa mRNA,^9^ which overlaps very well with m^6^A-SAC-seq results. Note that eTAM-seq enables site-specific and quantitative sequencing of m^6^A with as few as 10 cells,^9^ which requires much lower input RNA than other existing quantitative sequencing methods. Similar to the deamination principle in eTAM-seq but employing chemical reactions instead, Liu et al. developed glyoxal and nitrite-mediated deamination of unmethylated adenosines (GLORI) to quantitatively map m^6^A at base precision.^34^ GLORI maps m^6^A methylomes transcriptome-wide in mouse and human cells, revealing clustered m^6^A dynamics with information on site distribution and modification stoichiometry. The current version requires several hundred nanograms of RNA as the starting material, but further improvements should be able to lower the input amount substantially.

m^6^A-SAC-seq has two advantages over deamination-based methods such as eTAM-seq^9^ and GLORI:^34^ (i) only the m^6^A sites show mutation signatures, which preserves the sequence complexity and allows more accurate sequence mapping; (ii) the positive readout of m^6^A significantly reduces sequencing costs. A main limitation is the sequence context preference of the MjDim1 enzyme. Calibration probes are highly recommended for each sequencing library to reveal modification stoichiometry at different sequence contexts, since MjDim1 does exhibit sequence preference toward the GA motif. Note that while m^6^A-SAC-seq requires much less sequencing depth compared with eTAM-seq and GLORI, sequence bias needs to be calibrated. Taken together, m^6^A-SAC-seq, eTAM-seq, and GLORI currently serve as the three quantitative mapping tools targeting m^6^A (Table S1).

### Pseudouridine (Ψ)

Following m^6^A, pseudouridine (Ψ) is the second most abundant mRNA modification in mammalian mRNA, with ∼0.2% Ψ/U levels measured by mass spectrometry. Ψ modifications are known to distribute broadly in abundant noncoding RNAs, such as rRNA, tRNA, and small nuclear RNA (snRNA). Ψ is also known to exist in mammalian mRNA; however, sequencing Ψ within low-abundance mRNA was challenging using traditional experimental methods. Based on the chemical reaction with *N*-cyclohexyl-*N*′-(2-morpholinoethyl) carbodiimide methyl-*p*-toluenesulfonate (CMC) to yield CMC-modified Ψ and subsequent RT truncation signatures induced during reverse transcription,^35−37^ several next-generation sequencing methods were developed to map the transcriptome-wide distribution of cellular Ψ modifications, including Pseudo-seq,^35^ Ψ-seq,^36^ and PSI-seq.^37^ The RT truncation signatures are difficult to detect, especially at low- and medium-modified sites. Only modest numbers of Ψ sites were identified previously in mammalian mRNA with a low overlap among different data sets. An azide-modified CMC was applied to enrich Ψ-containing mRNA fragments in CeU-seq,^38^ with many more Ψ sites detected, but this method could not reveal Ψ stoichiometry transcriptome-wide. HydraPsi-seq,^39^ a method that relies on the resistance of pseudouridine toward hydrazine/aniline cleavage, was also reported to map Ψ modifications in yeast mRNA, but it cannot reveal Ψ stoichiometry either. The lack of a reliable method to map Ψ transcriptome-wide has been a bottleneck in functional investigations of Ψ in mRNA and other low-abundance RNA species.

We reported bisulfite-induced deletion sequencing (BID-seq)^2^ to quantitatively map Ψ at single-base resolution in 2022. Inspired by Khoddami et al., who reported RBS-seq (a modification of RNA bisulfite sequencing),^40^ a modified version of RNA bisulfite sequencing that enables the simultaneous detection of m^5^C, Ψ, and m^1^A at single-base resolution transcriptome-wide. A key discovery in RBS-seq was the observation of Ψ-dependent deletion signatures generated by a Ψ-bisulfite adduct during RT.^40,41^ Fleming et al. further investigated the chemical products^41^ generated at Ψ-modified sites after the bisulfite reaction in RBS-seq and identified two main adducts that were shown to induce the opening of the ribose ring and cause deletion signatures during reverse transcription. *N*^1^-methylpseudouridine (m^1^Ψ) in mRNA vaccines was shown to react similarly with bisulfite to yield ribose-opening products as well.^42^

**Figure 3:**
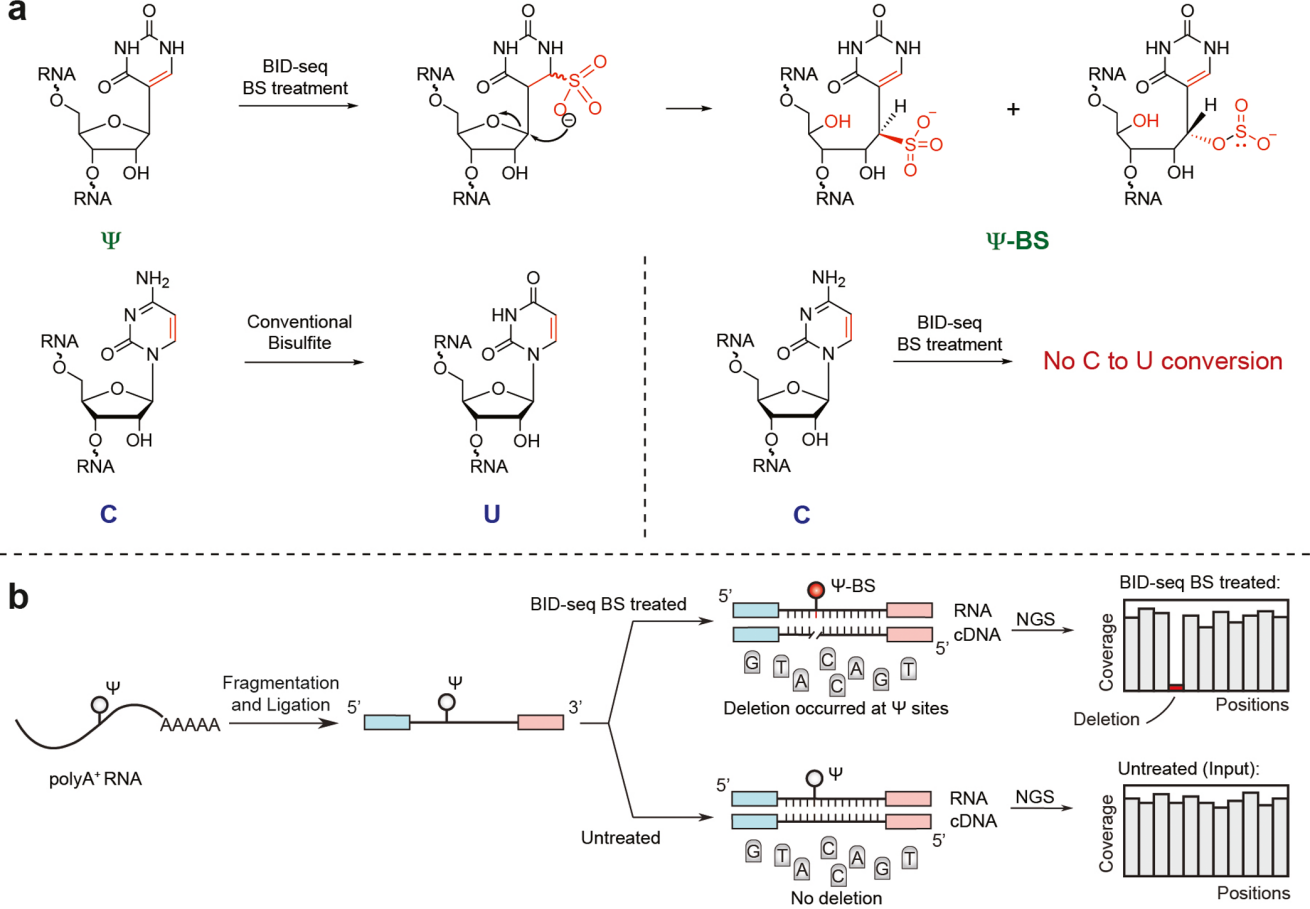
Base-resolution quantitative BID-seq for mapping pseudouridines in mammalian mRNA. (a) BID-seq bisulfite selectively reacts with Ψ sites but does not affect other RNA modifications or unmodified bases. (b) The brief BID-seq pipeline built on the NGS platform induces deletion signatures at internal Ψ sites.

RBS-seq still only uncovered very limited numbers of Ψ sites with weak signatures, mainly because of the conversion ratio of Ψ into Ψ-bisulfite adducts under conventional bisulfite reaction conditions. In the conventional bisulfite reaction, the protonation at the N3 position of cytosine typically requires an acidic pH, which facilitates the attack of BS to the C6 position to generate the C-BS adduct and subsequent deamination. We reasoned that the acidic condition is not optimal for the formation of Ψ-bisulfite adducts critical to the deletion signature during RT. We hypothesized that a neutral pH could not only enhance the production of Ψ-BS adducts but also inhibit C-to-U conversion to avoid reduced sequence complexity, achieving high Ψ detection sensitivity and accuracy^2^ (Figure 3a).

Indeed, when testing the synthetic RNA oligo probes of AGΨGA versus AGUGA and AGCGA, the matrix-assisted laser desorption/ionization-time-of-flight (MALDI-TOF) MS measurement clearly showed that bisulfite offered an almost quantitative conversion of Ψ to Ψ-BS adducts under neutral conditions,^2^ but the unmodified uridines were not affected after bisulfite treatment and desulphonation. No detectable C-to-U conversion was observed. Furthermore, we systematically screened all commercially available RT enzymes and self-made evolved RTs and observed that SuperScript IV reads out Ψ-BS adducts as deletion signatures with the highest deletion ratios among all known RTs.^2^ We then constructed BID-seq libraries using the NGS platform (Figure 3b). With the fully modified Ψ site within a synthetic oligo containing NNΨNN, 232 out of 256 motifs in NNΨNN gave deletion ratios over 50% at the Ψ sites after bisulfite treatment, with nearly all motifs showing >25% deletion ratios. We further validated 42, 53, and two known Ψ sites in HeLa 18S, 28S, and 5.8 rRNAs, respectively, without any false positives observed. BID-seq was applied to mRNA samples from human cell lines and mouse tissues, uncovering thousands of Ψ sites with stoichiometric information at each modified site.^2^

Thirteen pseudouridine synthase (PUS) enzymes are encoded in the human genome, with several specific PUS enzymes reported to install Ψ modifications in human mRNA.^43^ The quantitative feature of BID-seq enabled us to monitor Ψ stoichiometry change in PUS-depleted cells versus the control, and we found that mRNA Ψ sites could be either installed by a single specific PUS enzyme or by multiple PUS proteins.^2^ BID-seq revealed more than 100 Ψ-modified stop codons in 12 mouse tissues and confirmed the role of Ψ in promoting stop codon readthrough *in vivo*.^2^ Collectively, BID-seq set the stage for functional and mechanistic investigation of Ψ in low-abundance mRNA. In 2023, a similar approach, PRAISE, was also reported.^44^ In a further optimized BID-seq protocol, we achieved even lower background deletions at unmodified uridines after bisulfite treatment and uncovered around 8500 Ψ sites starting from as little as 10 ng poly(A) RNA from mouse embryonic stem cells (mESC;^45^Table S2).

### 5-Methylcytosine (m^5^C)

m^5^C exists in diverse RNA species including rRNA, tRNA, mRNA, and various noncoding RNAs. The antibody-based mapping method for RNA m^5^C, such as m^5^C-RIP-seq^46^ and 5-azacytidine-mediated RNA immunoprecipitation (Aza-IP),^47^ could provide neither single-base resolution nor m^5^C stoichiometry information, while miCLIP^48^ requires overexpression of the mutant enzyme. In recent years, BS-seq has been increasingly utilized to examine m^5^C modifications, with several commercial RNA BS conversion kits available, including the EZ RNA Methylation Kit from Zymo Research and the Methylamp RNA BS Conversion Kit from Epigentek. Using BS-seq, recent studies^49−52^ have revealed that m^5^C modification in mRNA and its regulator proteins impacts diverse cellular functions and plays crucial roles in development and cancer. However, the exact level and stoichiometry of m^5^C on mRNA have been a subject of debate due to the absence of a sensitive, robust, and quantitative sequencing method. Discrepancies were observed when conventional BS-seq was applied to low-abundance mRNA, with some studies detecting thousands of m^5^C sites in mRNAs^53^ while other studies discovered only a few sites.^54^ More recent studies have reported only a few hundred m^5^C sites in human and mouse transcriptomes using an improved bisulfite sequencing method and a more stringent computational approach.^50,51^ These inconsistent findings have raised the need to develop more sensitive and robust methods for identifying and quantifying real m^5^C sites in mRNA.

**Figure 4:**
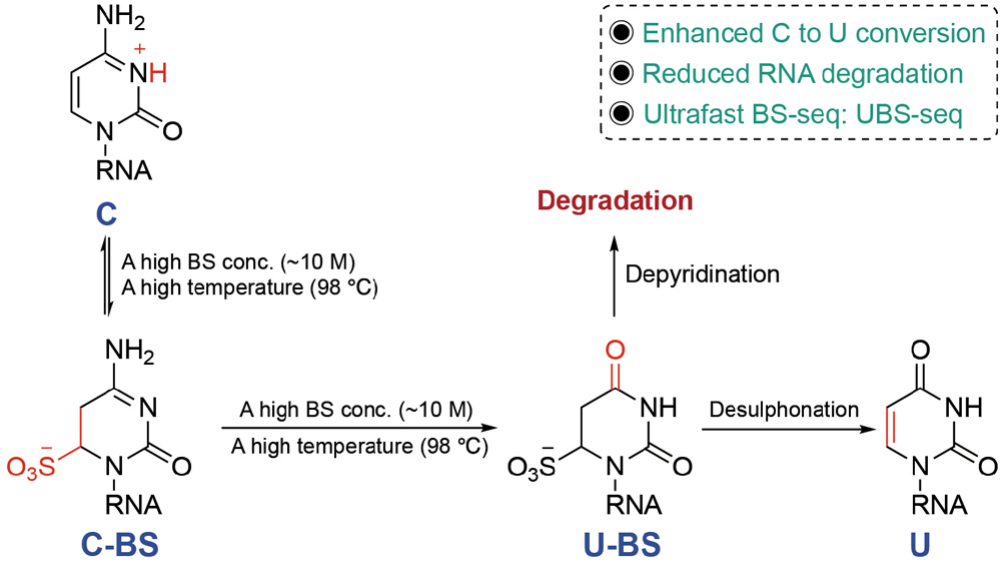
Chemistry principle of UBS-seq, a base-resolution approach for m^5^C quantification in mammalian mRNA.

A major challenge for RNA m^5^C BS sequencing has been the high false positive rates or high background caused by incomplete C-to-U conversion due to reduced reaction temperature and reduced reaction time to avoid severe RNA degradation. The reduced temperature is also ineffective in denaturing local secondary structures of highly structured RNAs, leading to further reduced C-to-U conversion at structured regions. Mechanistically, two competing pathways exist in bisulfite conversion of RNA, with one giving the desired C-to-U conversion and the other leading to the undesired RNA degradation. The protonated N3 nitrogen under acid conditions facilitates cytosine’s reaction with BS to give the C-BS adduct, which is converted to U-BS adduct by deamination. Subsequent desulphonation of the U-BS adduct under basic conditions generates U, completing C-to-U conversion. Alternatively, the U-BS adduct may undergo spontaneous depyrimidination to cause DNA degradation. To improve C-to-U conversion efficiency and reduce RNA damage, we developed ultrafast BS sequencing (UBS-seq)^3^ using a new recipe with a high BS concentration (∼10 M) and a high reaction temperature (98 °C; Figure 4). Applying UBS-seq to highly structured rRNA as a model, we showed that the average detected fraction for the two known m^5^C sites was >95%, while no false positive site was detected when a 5% cutoff for the unconverted rate of C was used. This new approach allowed detection of m^5^C stoichiometry in highly structured RNA species and outperformed all the reported BS conditions in terms of lower background and higher sensitivity in detecting real m^5^C sites in structured rRNA.^3^

When UBS-seq was applied to polyA^+^-enriched RNA from HeLa and HEK293T cell lines, input mRNA as low as 10–20 ng could yield transcriptome-wide m^5^C quantification, identifying 2723 and 2404 m^5^C sites with a modification fraction ≥5%, respectively.^3^ The quantitative nature of UBS-seq allowed us to reveal sequence motifs of m^5^C sites in mRNA and assign NSUN2 as the main m^5^C methyltransferase that installs ∼90% m^5^C sites to HeLa mRNA. To further validate the detected m^5^C sites with low modification fractions, with NSUN2 and NSUN6 as potential mRNA m^5^C “writer” proteins, we conducted rescue experiments by transfecting the corresponding methyltransferase plasmids back to the HeLa cells with NSUN2 or NSUN6 depletion and sequenced the isolated poly(A)-tailed RNA. Indeed, we observed that the decreased m^5^C fractions in the depleted strains were mostly rescued, further confirming that these lowly modified m^5^C sites are real. In addition, our results showed that m^5^C sites deposited by NSUN2 but not by NSUN6 are enriched in 5′-UTR regions in both HeLa and HEK293T mRNA, suggesting that m^5^C modification or its binding proteins may be involved in regulating mRNA translation.^3^ This new method and the data sets will aid future functional investigations on RNA m^5^C (Table S3).

### DAMM-seq to Map Base Methylations at Watson–Crick Base Pairing Interface

**Figure 5:**
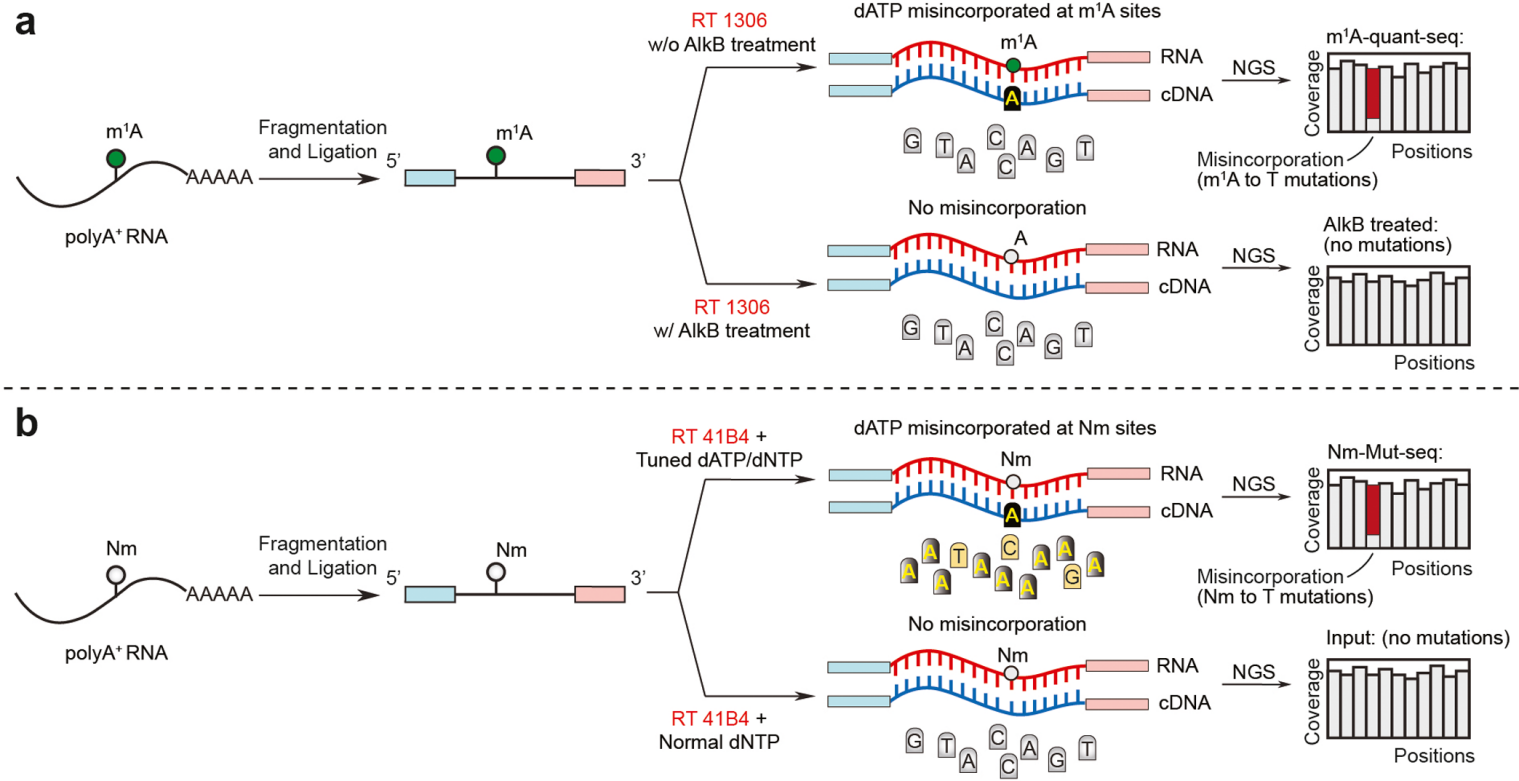
Quantitative base-resolution sequencing technology for m^1^A and 2′-*O*-methylation modifications in mammalian mRNA. (a) The brief m^1^A-quant-seq pipeline built on NGS platform induces a major A → T misincorporation signature at internal m^1^A sites. (b) The brief N_m_-Mut-seq pipeline built on the NGS platform induces A → T or C → T or G → T misincorporation signatures at internal A_m_, C_m_, and G_m_ sites.

We and others previously detected *N*^1^-methyladenosine (m^1^A) at ∼0.02% m^1^A/A abundance in mammalian poly(A)-tailed RNA.^55,56^ Different from m^6^A and Ψ, which cannot naturally induce misincorporation signatures during RT, the methyl group at the N1 position of the m^1^A base can disrupt base pairing and induce misincorporation in the presence of many commercially available RT enzymes, such as HIV RT, AMV RT, SuperScript II RT, and SuperScript IV RT. The mutation signals tend to be weak with notable RT stops observed. The TGIRT-based m^1^A-MAP displays excellent performance in m^1^A site detection in tRNA, but the low turnover of the TGIRT enzyme impedes the actual application of m^1^A-MAP for longer RNAs.^57^ We and our collaborators set up an evolution platform to evolve engineered RT enzymes for efficient readthrough of m^1^A with high rates of mutation signatures. We developed a fluorescence-based RT evolution platform for the direct evolution of RT enzymes, to select enzymes that give high RT misincorporation ratios of any given type of RNA modification.^10^ We started with HIV RT and identified RT1306, an evolved HIV RT to give robust readthrough and high misincorporation rates at m^1^A sites. Taking advantage of the evolved RT1306 and the AlkB-mediated demethylation at m^1^A sites as controls, we developed m^1^A-quant-seq^10^ (Figure 5a, which uncovered several hundred m^1^A sites in human polyA-tailed RNA, with stoichiometric information (Table S3). m^1^A-quant-seq also uncovered an array of mRNAs and lncRNAs containing highly modified m^1^A sites (above 30% modification stoichiometry),^10^ such as MALAT1, mt-ND5, PRUNE, etc., which is consistent with the previous publications and suggested functional roles.^58^

During our studies of m^1^A in mammalian cytosolic tRNA and mitochondrial tRNAs, we noticed m^1^A methylation on nascent mitochondrial RNA (mt-RNA). Mitochondrial transcription is unique in human cells, with bidirectional transcription producing a long precursor mt-RNA that contains two mt-rRNA, 13 mt-mRNA, and 22 mt-tRNA serving as junctions within the mitochondrial polycistronic RNA. In addition to m^1^A methylations, other base methylations appear to also play roles in nascent mt-RNA processing. We therefore developed demethylation-assisted multiple methylation sequencing (DAMM-seq),^4^ which starts with ∼10 ng of input RNA and quantitatively maps not only m^1^A but also *N*^3^-methylcytidine (m^3^C), *N*^1^-methylguanosine (m^1^G), and *N*^2^,*N*^2^-dimethylguanosine (m^2^_2_G) in mitochondrial polycistronic RNA. These modifications all block the Watson–Crick base pairing interface and exist in limited sequence motifs in tRNAs; HIV RT already reads through these known motifs well and induces high misincorporation rates for quantitative stoichiometry determination.^4^

DAMM-seq utilizes the misincorporation ratios obtained at each methylated site for estimating the methylation stoichiometry, enabling the quantitative characterization of m^1^A, m^1^G, m^3^C, and m^2^_2_G in nascent mt-RNAs.^4^ As an application example of quantitative DAMM-seq, we applied DAMM-seq to sequence mitochondrial polycistronic RNA under different cellular treatments, particularly ALKBH7 depletion and overexpression. DAMM-seq sensitively monitored the methylation level changes at methylated m^1^A, m^1^G, m^3^C, and m^2^_2_G sites within mitochondrial polycistronic RNA and uncovered an ALKBH7 demethylation effect at m^1^A and m^2^_2_G sites within pre-tRNA regions of mt-Leu1 and mt-Ile, respectively. This study identified ALKBH7 as an RNA demethylase regulating mitochondrial RNA processing and mitochondrial activity.^4^ DAMM-seq serves as an effective approach for the quantitative investigation of multiple RNA methylations within nascent RNA species of an input RNA amount as low as 10 ng (Table S3). A similar method named PANDORA-seq was also reported around the same time.^59^

### 2′-*O*-methylation (N_m_)

Among the expanding list of functionally relevant post-transcriptional RNA modifications, methylation of the 2′-OH of an RNA ribose (2′-*O*-methylation, N_m_) is both abundant and unique. 2′-*O*-methylation is one of the most common RNA modifications present in many cellular RNAs, such as tRNA, rRNA, and small nuclear RNA (snRNA). It also exists in mammalian mRNA and is about 10-fold less abundant compared to m^6^A. Unlike base modifications, which are specific for one of the four RNA bases, N_m_ methylation occurs at the 2′-OH position of each ribonucleoside. N_m_ modifications can modulate the secondary structure of rRNA and tRNA and affect mRNA stabilization and translation.^60,61^ While functional roles of N_m_ modifications on abundant tRNAs and rRNAs have been well documented, studies of their impacts on lower-abundance RNAs, such as mRNAs and long noncoding RNAs (lncRNAs), have been hampered by the lack of effective antibodies and robust sequencing methods that map N_m_ at base resolution.

Previous base-resolution approaches using NGS have been successful at identifying 2′-*O*-methylation in highly abundant RNA.^62−66^ However, most of these methods cannot provide stoichiometric information at the modified sites. RiboMethSeq^65^ is the only quantitative method developed thus far, but very deep sequencing depth is required for confident N_m_ detection in low-abundance RNAs and at low-stoichiometry N_m_ sites. In addition, most current N_m_ mapping methods mainly rely on RT truncation signatures induced by chemical-assisted cleavage, which may include substantial false positives arising from RT stops at structured regions in RNA or unmodified RNA ends from fragmentation biases or RNA processing. Compared with RNA truncation signatures, modification-dependent misincorporations as readouts are generally more reliable in modification detection and quantification. However, since N_m_ modification occurs on the ribose and does not directly modulate the Watson–Crick base pairing interface during reverse transcription, all known reverse transcriptases (RT) are unable to induce misincorporations, which has precluded the use of mutation-based analysis methods for N_m_ mapping. Leveraging the fluorescence-based RT evolution platform to generate RTs that are mutagenic at specific RNA modification of interest,^8^ we speculated that evolved RTs might be selected to generate misincorporations opposite of N_m_-modified bases.

Indeed, a new HIV RT variant, RT-41B4, was selected to yield notable misincorporation signatures at N_m_-modified sites. In the presence of adjusted dATP/dNTP ratios during RT, we developed N_m_-Mut-seq (an RT-mutation-based N_m_ mapping method)^11^ that maps C_m_, G_m_, and A_m_ methylations at single-base resolution with stoichiometry information (Figure 5b). To validate N_m_-Mut-seq, we mapped almost all known C_m_, G_m_, and A_m_ sites on human rRNAs with high misincorporation rates and without observing any false positives at unmodified sites or other rRNA modifications.^11^ Applying N_m_-Mut-seq to HepG2 cellular mRNA, we uncovered around 1000 C_m_, G_m_, and A_m_ sites, a subset of which were validated by multiple orthogonal methods,^11^ further confirming the fidelity of the method. Fibrillarin (FBL) was shown to be a main N_m_ “writer” protein for HepG2 mRNA, and the methylation is guided by snoRNAs that interact with the target mRNAs. Note that the current N_m_-Mut-seq still requires ∼200–800 ng of input polyA^+^ RNA (Table S3); otherwise, the high PCR cycle numbers lead to dramatic PCR duplicates in the obtained NGS data. N_m_-Mut-seq, therefore, provides an effective method to not only detect N_m_ at base resolution in low abundant RNAs such as mRNA and lncRNA but also measure the stoichiometry of the modified sites transcriptome-wide.

### Internal *N*^7^-Methylguanosine (m^7^G)

m^7^G methylation is a well-known modification in the cap structure of the 5′ end of mammalian mRNA, which impacts diverse biological processes such as mRNA stability, splicing, nuclear export, and translation. Highly modified internal m^7^G sites were also found at cytoplasmic tRNA and 18S rRNA.^2^ However, whether m^7^G methylation could be deposited internally to mammalian mRNA remained unknown for a long time. In 2019, we and another group demonstrated the existence of internal m^7^G modification in mammalian mRNA and miRNA.^12,67^ We reported the antibody-based m^7^G-MeRIP-seq and a single-base resolution m^7^G sequencing method m^7^G-seq^12^ (Table S3).

Using m^7^G-MeRIP-seq, we identified METTL1 as the main “writer” protein that installs mRNA internal m^7^G. m^7^G is positively charged and could be converted into a reduced state in the presence of sodium borohydride; the subsequent heating under acidic conditions leads to depurination at the reduced m^7^G site, yielding an abasic site (AP site).^12^ After screening different commercially available RT enzymes, we found that the abasic site generated at the m^7^G site could be read as RT misincorporation signals using HIV RT; the abasic sites could be further captured and enriched by biotin-tagged hydrazine to yield a higher misincorporation rate. Overall, m^7^G-seq includes three libraries for each sequencing library: zero mutations at internal m^7^G sites in the “Input” library, a moderate mutation rate induced at abasic sites in “Before pulldown” libraries, and a high mutation rate induced at biotin-enriched abasic sites in “Pulldown” libraries.^12^ The base-resolution m^7^G-seq confirmed the presence of mRNA internal m^7^G methylations in cancer cell lines. Note that a recent study revealed that quaking proteins (QKIs) bind to internal m^7^G-modified mRNAs with GA-rich motifs, as the first reported internal m^7^G “reader” proteins,^68^ suggesting functional roles of mRNA internal m^7^G methylations in mammals.

**Figure 6:**
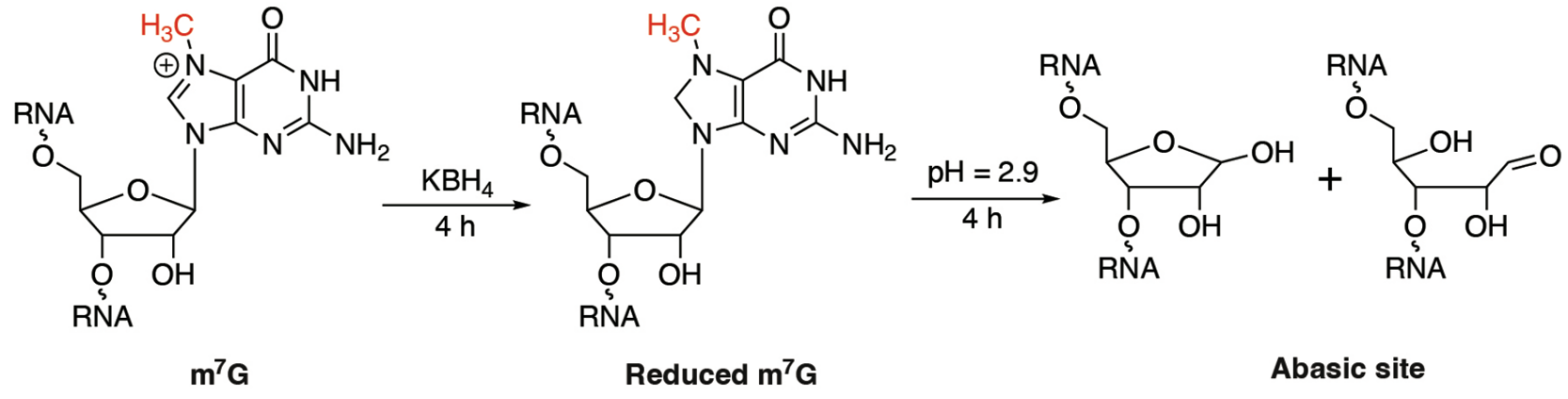
Chemistry principle for base-resolution quantitative mapping of internal m^7^G methylations.

Besides the role of METTL1 as the 'writer' protein for mRNA internal m^7^G installation, METTL1 is well-known to mediate m^7^G methylation at position 46 of cytoplasmic tRNA, and this methylation has been shown to link with cancer progression and tumorigenesis,^69−71^ requiring a quantitative method for monitoring tRNA m^7^G dynamics in pathological processes. Based on the chemical principle in m^7^G-seq, we further optimized it to be more quantitative (Figure 6), or m^7^G-quant-seq,^13^ without the need for the biotin pulldown enrichment. In m^7^G-quant-seq, we performed the depurination at a more acidic pH (∼2.9) under mild heating. The internal m^7^G sites could be almost completely converted to abasic sites when extending the incubation time of both KBH_4_ reduction and depurination.^13^ Note that the current m^7^G-quant-seq requires ∼200 ng of cellular small RNA as input, to avoid high PCR cycle number and the consequent PCR duplicates in NGS^13^ (Table S3).

## CONCLUSION AND PERSPECTIVES

In summary, the lack of reliable sequencing methods to map modifications on mammalian mRNA has hampered functional investigations of these modifications. Applying chemical and biochemical knowledge we have developed a set of methods to map mRNA modifications at base resolution with modification stoichiometry information. These sequencing methods allow base resolution mapping of most major mRNA modifications often with limited input sample requirements, particularly m^6^A-SAC-seq, BID-seq, UBS-seq, and DAMM-seq, which could start with ∼2 ng, ∼10 ng, ∼10–20 ng, and ∼10 ng input RNA, respectively.^1−4^ They could be used to uncover dynamic changes in modification stoichiometry during biological processes. The use of calibration spike-in probes could further enhance the accuracy of stoichiometry determination. For m^6^A-SAC-seq, the use of calibration probes is highly recommended to ensure accurate modification stoichiometry quantification at different sequence contexts because of the sequence context preference of the MjDim1 enzyme. Spike-in probes are not necessary for UBS-seq, since the chemical treatment displays an extremely high conversion ratio with no noticeable preference in sequence context around unmethylated cytidines. For other methods mentioned in this Account, such as BID-seq, N_m_-Mut-seq, and m^1^A-quant-seq,^2,10,11^ calibration probes are recommended for reproducing observations on modification stoichiometry when using different batches of engineered or commercially available RT enzymes. These RT enzymes may have sequence preferences. The application scope of these methods goes beyond mRNA and could also be readily applied to other RNA species, such as nuclear nascent RNA, caRNA, cellular small RNA, cell-free RNA, etc.

The application of quantitative m^6^A-SAC-seq has revealed dynamics of m^6^A methylation stoichiometry during cell differentiation and characterized a number of cell-state-specific m^6^A sites.^1^ Quantitative BID-seq confirmed Ψ-modified stop codons within mammalian mRNAs, as *in vivo* on/off switches for converting nonsense codons into sense codons.^2^ In this Account, we emphasized the quantitative feature of our recently developed sequencing methods, and these developments may lead to large-scale high-dimensional maps on quantifying the dynamics of RNA modification stoichiometry in diverse biological processes, instead of only depicting the occurrence of an RNA modification type. Furthermore, the high conversion ratios of Ψ to Ψ-BS in BID-seq and C to U in UBS-seq and the ease of use, respectively, may make both BID-seq and UBS-seq common methods for future detection of Ψ and m^5^C modifications in mRNA and other RNA species. There are advantages and disadvantages for the base-resolution methods to map m^6^A, namely, m^6^A-SAC-seq, eTAM-seq, and GLORI.^1,9,34^ While m^6^A-SAC-seq exhibits sequence bias, it is a method that reads out m^6^A as a mutant, thereby maintaining the sequence complexity and notably reducing sequencing costs.

There is still plenty of space to optimize these methods to be more sensitive and more accurate and use less starting material. Single-cell level sequencing could reveal heterogeneity information and help further classify cell types. A future challenge is to sequence multiple modifications on the same RNA molecule. How compatible these methods are with each other and how practical to run multiple reactions before sequencing are questions that will need to be addressed. Chemical or biochemical labeling of specific modifications, coupled with long-read sequencing platforms such as nanopore or PacBio, may offer an attractive solution moving forward to simultaneously map multiple modifications on a single RNA molecule.

## Supporting Information Available

A summary of published sequencing methods for mapping mRNA modifications (PDF)
