# Supplementary material for: Base-Resolution Sequencing Methods for Whole-Transcriptome Quantification of mRNA Modifications
Source: Acc Chem Res. 2023 Dec 11;57(1):47–58. doi: 10.1021/acs.accounts.3c00532 (PMC10765377; doi:10.1021/acs.accounts.3c00532)
Supplement: Supplementary file 1 — ar3c00532_si_001.pdf [file ar3c00532_si_001.pdf]

Supplementary Information

Base-resolution sequencing methods for whole-transcriptome quantification of messenger RNA modifications

Li-Sheng Zhang[1,2,3,4,#], Qing Dai[1,2,#], Chuan He[1,2,*]

[1]Department of Chemistry, The University of Chicago, Chicago, IL, 60637, USA.

[2]Howard Hughes Medical Institute, The University of Chicago, Chicago, IL, 60637, USA.

[3]Department of Chemistry, The Hong Kong University of Science and Technology (HKUST), Kowloon 999077, Hong Kong SAR, China.

[4]Division of Life Science, The Hong Kong University of Science and Technology (HKUST), Kowloon 999077, Hong Kong SAR, China.

[#] These authors contributed equally.

*To whom correspondence should be addressed (chuanhe@uchicago.edu).

Table S1. A summary of published whole-transcriptome m^6A profiling methods.

Method	Base-resolution?	Quantitative?	Input RNA amount	Covering all motifs?	Direct m^6A detection	Antibody free?
m^6A-MeRIP-seq	No	No	2–400 µg mRNA	Yes	No	No
miCLIP-seq	Yes	No	20 µg mRNA	Yes	No	No
m^6A-LAIC-seq	No	No	~4 µg mRNA	Yes	No	No
m^6A-REF-seq & MAZTER-seq	Yes	Yes	100 ng mRNA	No	No	Yes
DART-seq	Yes	No	10 ng ~1 µg total RNA	No	No	Yes
m^6A-SEAL	No	No	5 µg mRNA	Yes	No	Yes
m^6A-label-seq	Yes	No	5 µg total RNA	Yes	Yes	No
m^6A-SAC-seq	Yes	Yes	~2 ng mRNA	Yes	Yes	Yes
eTAM-seq	Yes	Yes	RNA from as few as 10 cells	Yes	No	Yes
GLORI	Yes	Yes	200 ng mRNA	Yes	No	Yes

Table S2. A summary of published whole-transcriptome Ψ profiling methods.

Method	Base-resolution?	Quantitative?	Input RNA amount	Pulldown enrichment?	Detection signature	Chemicals applied	Applicable to mammalian mRNA?
Ψ-seq	Yes	No	Not specified	No	RT stop	CMC	Yes
Pseudo-seq	Yes	No	mRNA from 2~10 µg total RNA	No	RT stop	CMC	Yes
PSI-seq	Yes	No	3 µg mRNA	No	RT stop	CMC	No
CeU-seq	Yes	No	10 µg mRNA	Yes	RT stop	CMC	Yes
RBS-seq	Yes	No	5 µg mRNA	No	Deletion	Bisulfite	Yes
HydraPsi-seq	Yes	Yes	10~50 ng mRNA	No	Resistant to hydrazine/aniline cleavage	Hydrazine/ aniline	No
BID-seq	Yes	Yes	10 ng mRNA	No	Deletion	Bisulfite	Yes
PRAISE	Yes	Yes	500 ng mRNA or total RNA	No	Deletion	Bisulfite	Yes

Table S3. A summary of our recently developed sequencing methods for whole-transcriptome profiling of m^5C, m^1A, 2'-*O*-methylation (Nm), and internal m^7G.

**Method**	**Base-resolution?**	**Quantitative?**	**Input RNA amount**	**Pulldown enrichment?**	**Detection signature**	**Engineered RT applied?**	**Which base is to be detected?**
UBS-seq	Yes	Yes	10~20 ng mRNA	No	Mutation	No	m^5C
DAMM-seq	Yes	Yes	~10 ng RNA	No	Mutation	No	m^1A, m^3C, m^1G, and m^2_2G
m^1A-quant-seq	Yes	Yes	~100 ng mRNA	No	Mutation	Yes	m^1A
Nm-Mut-seq	Yes	Yes	200~800 ng mRNA	No	Mutation	Yes	2'-*O*-methylation
m^7G-seq	Yes	No	4~6 µg mRNA	Yes	Mutation	No	Internal m^7G
m^7G-quant-seq	Yes	Yes	~200 ng cellular small RNA	No	Mutation	No	Internal m^7G
